# A Two-Stage Localization and Refinement Neural Network Structure for Data-Efficient Microbleed Detection

**DOI:** 10.3390/brainsci16020207

**Published:** 2026-02-10

**Authors:** Lukas Rau, Oliver Granert, Nils G. Margraf, Stephan Schneider, Ulf Jensen-Kondering

**Affiliations:** 1 Faculty of Computer Science and Electrical Engineering, HAW Kiel, 24149 Kiel, Germany; 2 Department of Neurology, University Hospital Schleswig-Holstein, Campus Kiel, 24105 Kiel, Germany; o.granert@neurologie.uni-kiel.de (O.G.); n.margraf@neurologie.uni-kiel.de (N.G.M.); 3 Department of Business Information Systems, Faculty of Business Management, HAW Kiel, 24149 Kiel, Germany; stephan.schneider@haw-kiel.de; 4 Department of Neuroradiology, University Hospital Schleswig-Holstein, Campus Lübeck, 23538 Lübeck, Germany; 5 Department of Radiology and Neuroradiology, University Hospital Schleswig-Holstein, Campus Kiel, 24105 Kiel, Germany

**Keywords:** cerebral microbleed, convolutional neural network, deep learning, susceptibility-weighted imaging, U-Net

## Abstract

**Background/Objectives**: In medical diagnostics, (semi-)automatic detection of pathological structures in images is becoming increasingly important. In particular, detecting cerebral microbleeds (CMBs) poses a challenge in clinical practice because the process is time-consuming and prone to error. **Methods**: Compared to previous methods of (semi-) automatic CMB detection that rely on large training datasets, we propose a method that can be adapted with a small dataset while still performing well. We propose a workflow that uses a two-stage approach to detect cerebral microbleeds that can be trained with a small dataset. The first stage is a 3D U-Net that retrieves potential CMB locations in the SWI image volume. Then, a 3D convolutional neural network (CNN) is used for discrimination to distinguish between real CMB and CMB mimics. **Results**: Using a dataset of 15 MRI scans with 40 marked CMBs, we are able to achieve a sensitivity of 97.5%. **Conclusions**: We showed that it is possible to create a workflow with high sensitivity using only a few training samples, enabling smaller radiological facilities to train networks using their own datasets. Even though the workflow performs well on a small dataset, it still requires further testing with other larger datasets.

## 1. Introduction

**Figure 1:**
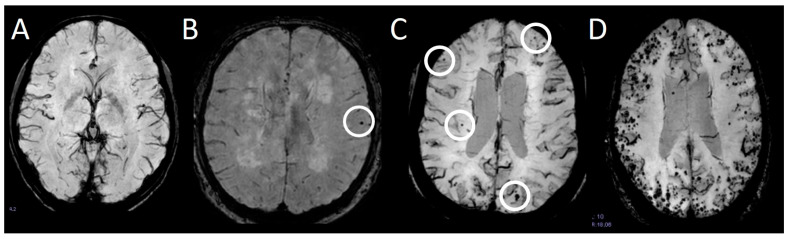
Blood sensitive sequences (susceptibility weighted imaging, SWI) demonstrating no CMB (**A**), a single CMB ((**B**), circle), a few cerebral microbleeds ((**C**), circles highlighting some CMBs), and extensive cerebral microbleeds (**D**).

Cerebral microbleeds (CMBs) are 2–10 mm small accumulations of blood in the brain parenchyma. They are defined as round or ovoid signal hypointensities on sequences sensitive to blood products, such as susceptibility-weighted images (SWI) or gradient echo (GRE) T2*-weighted images, and are typically not visualized on other sequences or other imaging modalities [1]. They have been associated with cognitive decline [2,3] and vascular diseases [4,5]. While single CMBs are mostly asymptomatic, the total number of CMBs predicts the risk of future symptomatic macro-hemorrhages [6]. CMBs are the hallmark of several diseases, the most common of which are cerebral amyloid angiopathy (CAA) [7] and hypertensive arteriopathy (HTN-A) [8]. Especially when reading SWIs, some typical mimics must be considered. A common problem encountered in daily practice is distinguishing a CMB from a venous blood vessel [9]. While a CMB is confined to a few adjacent slices, a blood vessel is continuously visible through multiple slices. Other mimics, such as hemorrhagic metastasis, diffuse axonal injury, or calcifications [9] or residuals from heart-lung machines during open heart surgery [10] must also be considered. In some locations, e.g., the anterior temporal lobes, it is more demanding to discern single CMBs because they are more prone to seemingly bleed into surrounding structures such as blood vessels and susceptibility artifacts caused by the bone-CSF border. Manual detection of CMBs, especially when there are many, as shown in Figure 1D, can be demanding and time-consuming, e.g., to assess the progression of the number of CMBs.

Furthermore, it is error-prone (“satisfaction of search”, “underreading” [11]) and is associated with great inter-rater variability. Thus, an automated and objective algorithm to detect and count CMB is desirable, especially in clinical routine or in a research setting if large patient cohorts are involved.

Different approaches have been employed in prior research, utilizing both semi-automated and fully automated systems (Table 1). All these approaches focus on increasing sensitivity and decreasing false positives. They, however, differ in the achieved sensitivity, the number of false positives per scan, and the number of scans used for training. Here, we propose an approach that combines advanced image preprocessing as proposed by Koschmieder et al. [12] and Suwalska et al. [13], with a 3D U-Net localization stage and a subsequent 3D CNN discrimination stage as proposed by Dou et al.

## 2. Materials and Methods

### 2.1. Methodology

**Figure 2:**
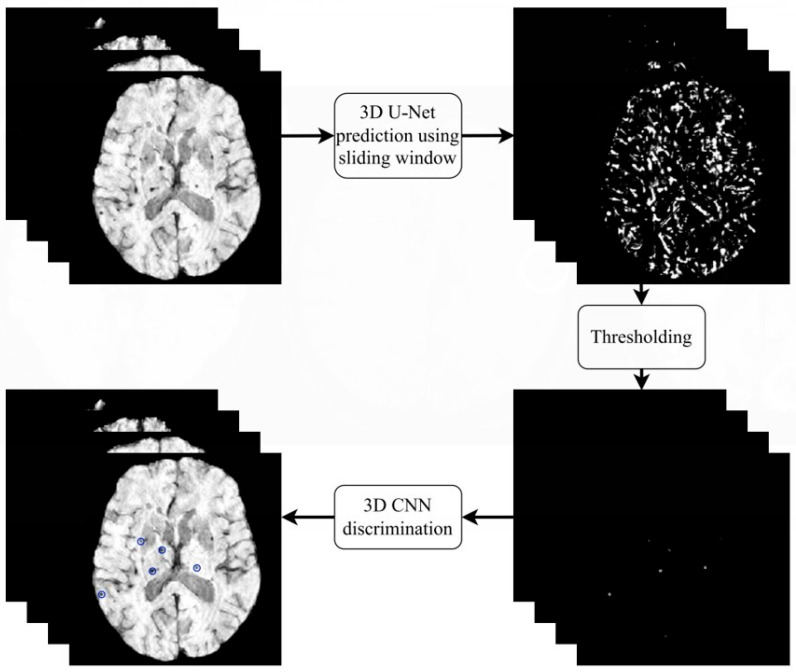
Proposed workflow with prediction and discrimination nets. Blue circles indicate detected CMBs from the output of the discrimination nets.

The general structure of the proposed workflow is shown in Figure 2. First, the scans are preprocessed and subsequently passed to the 3D U-Net. The 3D U-Net is applied using a sliding window strategy with a window size of 20 × 20 × 16 voxels and a stride of three for each axis. These prediction volumes are then stitched together by overlaying them based on their positions in the scan, forming a combined prediction volume. After that, we threshold the prediction volume to extract the possible CMB locations. These possible CMB locations are passed to the 3D CNN, which uses a 20 × 20 × 16 voxel window around the possible CMB location to predict the likelihood that this location is a real CMB. The workflow heavily relies on CNNs [13].

#### 2.1.1. 3D U-Net

**Figure 3:**
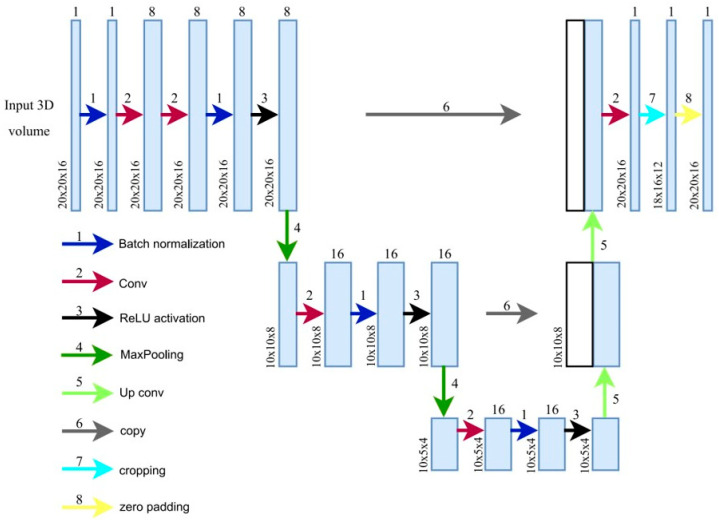
3D U-Net architecture.

**Figure 4:**
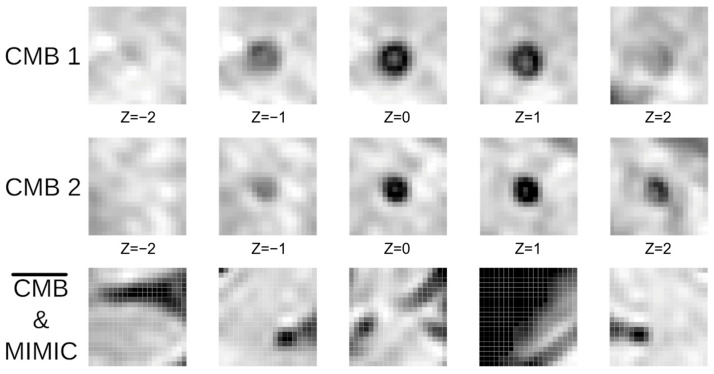
Two samples of different CMB sliced along the z-axis (rows CMB1 and CMB2) and negative examples, including CMB mimics, used for training the network weights (last row).

Marking CMB candidates and maximizing sensitivity are the main objectives of the first stage of our CMB detection process. We use a 3D U-Net (Figure 3) which takes an image of 20 × 20 × 16 voxels as input and outputs a prediction map of the same size, marking possible CMB locations. This, compared to the approach of Dou et al. [17], provides a larger network input size, thereby increasing the field of view. This extended view can help discriminate between blood vessels and microbleeds, since CMBs are mostly surrounded by regions that are less paramagnetic and thus appear bright on the scans (Figure 4). These long blood vessel structures can be identified with our larger field of view. Our larger sliding window enables faster processing by allowing larger strides in the CMB sliding window localization procedure. The convolutional layer applies a filter to the input volume and creates a feature map that sums up the detected features of the input. The batch normalization layer helps the 3D U-Net achieve better sensitivity while reducing training time and stabilizing gradients during training [23,24].

#### 2.1.2. 3D CNN

**Figure 5:**
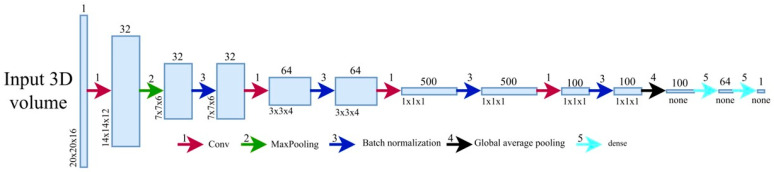
3D CNN architecture.

A 3D CNN is used in the second stage to discriminate CMBs from CMB mimics (see Figure 4). Our net uses a 20 × 20 × 16 input volume and predicts how likely it is for the center of the volume to be a CMB (Figure 5). The net structure is based on the architecture proposed by Dou et al. [17] but additionally uses some batch normalization layers to speed up the training time. These layers prevent undesired high weights from cascading through the neural network during training by normalizing the output of the activation function and then scaling and shifting this normalized output by two trainable parameters. This helps the network’s weight optimization during training achieve smoother, more predictive, and more stable gradient behavior [23,24]. The convolutional layers act as pattern detectors. Small kernels of different sizes stride over the input volumes, transforming them into feature maps. The MaxPooling layer helps create a down-sampled (pooled) feature map and adds translation invariance, so that a small shift in the CMB does not significantly affect the detection performance. After passing through multiple convolutional layers, the feature maps are then flattened, resulting in a single output representing the probability of a CMB presence at the center of the input volume.

### 2.2. Experiments

#### 2.2.1. Dataset and Preprocessing

**Figure 6:**
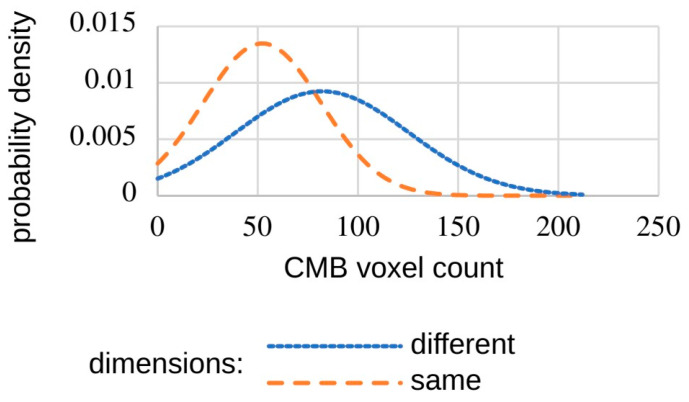
Distribution of voxel values from two different scans pre-normalization.

For the training and testing of our network architecture, we use a dataset provided by Dou et al. [19]. The dataset consists of 20 SWI scans from 20 different subjects with at least 1 and up to 13 annotated CMBs. However, some scans in this dataset have a different voxel matrix. If we use these images for training, the sensitivity of our neural networks decreases due to differences in CMB volume, as demonstrated in Figure 6. When only using the scans at the same resolution, the mean CMB voxel count is 52 with a standard deviation of 29.5. In comparison, the CMB mean voxel count in the other scans is 82 with a standard deviation of 43. This is why we use only 15 of the 20 datasets, all of which had 512 × 512 × 150 voxels, resulting in 40 marked CMBs in total.

This reduction in the training dataset does not affect our network’s performance, as it better shows the feasibility and performance of our workflow when trained and tested on a small dataset.

**Figure 7:**
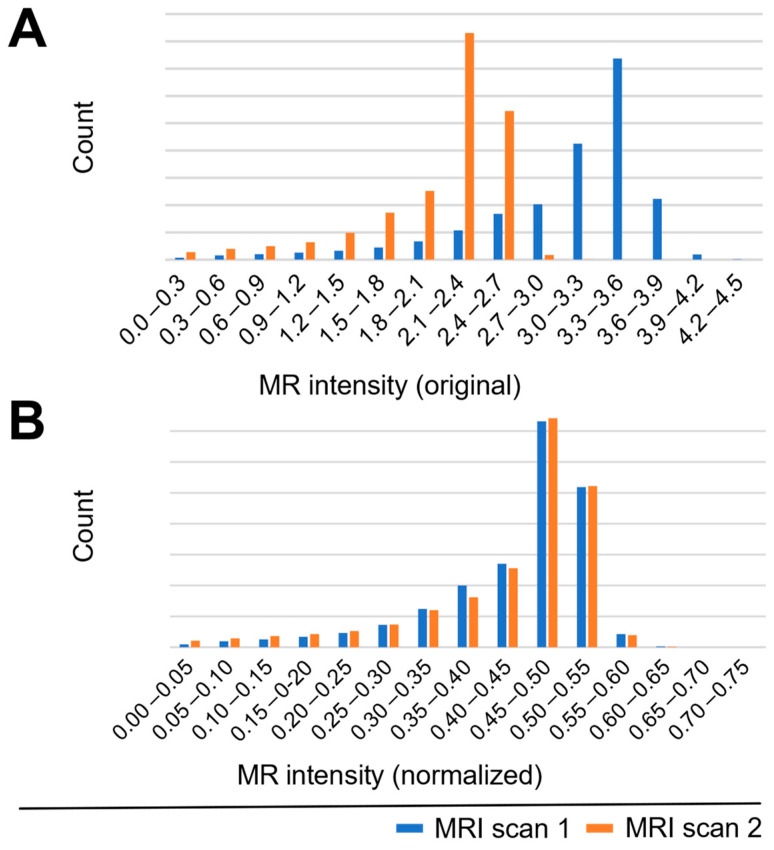
Distribution of voxel values from two different (blue and orange) scans (**A**) pre- and (**B**) post-normalization.

The first step in preprocessing is brain masking, which we perform using the HD-BET software (Version 2.0.1) [25]. Although this tool was not specifically designed for SWI scans, its performance is sufficient for this paper. To remove the low-frequency intensity non-uniformity, which is often present in image data, also known as gain field or bias, we use the “N4ITK” (Version 5.3), which is available through the Insight Toolkit of the National Institutes of Health [26]. In the last step, we normalize the intensity values of the data, similar to Koschmieder et al. [21], by detecting the peak h_p_ of the image intensity histogram from the MRI volume (except zero background intensity) and linearly map the data by

histnorm (*i*) = *i*/(2 ∗ h_p_)

where *i* is the image intensity before normalization. After this normalization, the histogram peak maps to an intensity of 0.5. Figure 7 demonstrates the effect of normalization on the histograms of two example scans. The normalization step effectively compensates for intensity variations across MRI acquisitions and improves the detection performance.

#### 2.2.2. Augmentation

Since deep learning tasks usually require a huge amount of data, we augment the data provided by Dou et al. [17]. Three typical augmentation methods were used to increase the amount of training data. The CMB patches were flipped, rotated 90 degrees in both directions, and shifted by one voxel position to maintain the CMB’s location at the center and incorporate the desired variety into the dataset.

#### 2.2.3. Models and Mimics

The architecture (Figure 4) is used in the training process of our 3D U-Nets. We train two of these 3D U-Nets with the same network structure and apply them in parallel for CBM detection. The first 3D U-Net is trained with a batch size of 20, whereas the second is trained with a batch size of 30. Although this approach is more time-consuming, we are able to achieve a higher sensitivity with a lower average count of false positives. The two prediction maps are normalized and added together to form a final prediction map for each validation scan. By combining these prediction maps, we put more emphasis on the points where the 3D U-Nets predict a CMB. This process achieves a higher sensitivity. For instance, if one 3D U-Net fails to detect a CMB, the other 3D U-Net potentially detects the missed CMB.

**Figure 8:**
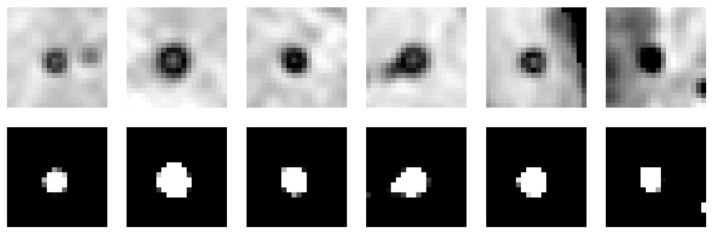
CMB and their corresponding 3D U-Net predictions.

After each training step, the 3D U-Nets use two entire scans as a validation set. This scheme corresponds to a leave-two-out cross-validation (LOTCV) with a sliding window strategy, using a stride size of three voxels in each dimension to create a prediction map for each validation set (Figure 8). A LOTCV can be seen as an extension of leave-one-out cross-validation (LOOCV).

To optimize the second-stage training of the 3D CNN, we applied a threshold of 0.15 to the combined U-Net predictions. While this does not affect sensitivity, it increases the false positive count. The coordinates of these false positives are saved in a scan-specific file. This approach was chosen because the 3D U-Net is highly sensitive to low-intensity foci but struggles to differentiate between circular microbleeds (CMB) and blood vessels of similar intensity. By incorporating these ‘mimics’ into the training set, the 3D CNN learns to identify 3D U-Net errors, enhancing its ability to discriminate between real CMBs and artifacts. The training workflow for the 3D CNN follows a similar protocol to the 3D U-Nets, utilizing data augmentation through rotation, flipping, and coordinate shifting, now supplemented by the identified mimics. We trained two 3D CNNs in parallel using different batch sizes of 20 and 30, respectively.

### 2.3. Hardware and Software

The training and testing of the composed model system were performed on a machine equipped with an AMD (Advanced Micro Devices, Inc., Santa Clara, CA, USA) Ryzen 7 5800x with 16-core parallel processing and 32 GB of RAM. The model system was created and executed with the Tensorflow framework (https://www.tensorflow.org/) (accessed on 20 July 2022).

## 3. Results

We train two 3D U-Nets with the same network architecture and apply them in parallel for CBM detection. The first 3D U-Net is trained with a batch size of 20, whereas the second is trained with a batch size of 30. Combining two different batch-sized 3D U-Nets improves the sensitivity, as shown in Table 2. This is especially valuable because of the small number of marked CMBs, where a higher sensitivity can be more valuable than a lower time of processing. We obtained a relatively high sensitivity with both solitary 3D U-Nets, which provides the opportunity to choose between a lower processing time or a higher sensitivity.

By thresholding the combined predictions at a value of 0.2, we obtain a first-stage sensitivity of 98.33% with 38.15 false positives per scan.

**Figure 9:**
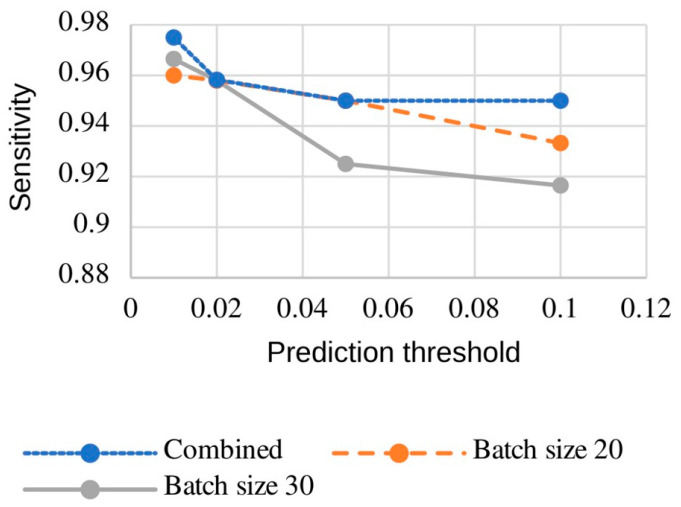
Influence of prediction threshold on the sensitivity across various 3D CNN training strategies and ensemble combinations.

We also train two 3D CNNs in parallel using different batch sizes of 20 and 30, respectively. The combination of two 3D CNNs in the second stage yields slightly higher sensitivity than either 3D CNN alone (see Figure 9).

**Figure 10:**
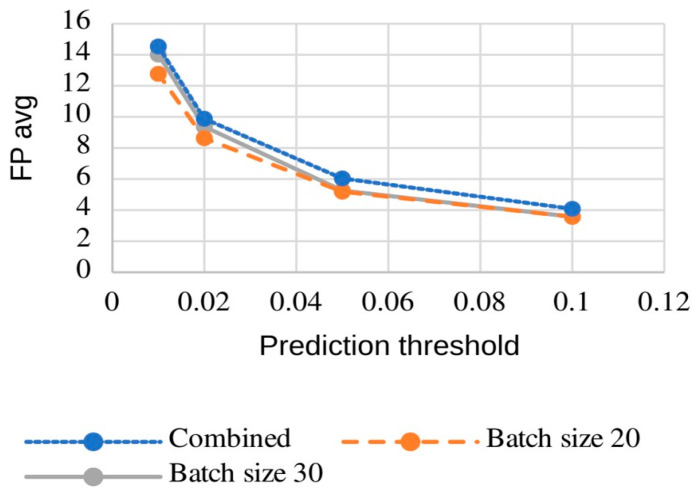
Influence of prediction threshold on the average false positive rate across various 3D CNN training strategies and ensemble combinations.

While combining the predictions from both models may not significantly increase sensitivity compared to the model trained with a batch size of 20, we nevertheless recommend this ensemble approach. The computational overhead is minimal, with parallel 3D CNN inference adding only a few seconds to the processing time. This combination leads to a marginal increase in false positives compared to the individual models (batch sizes 20 and 30) (see Figure 10).

Given our specific workflow and the extreme class imbalance—where over 99% of voxels are negatives—we have opted to present our results using “sensitivity” and “average false positive rate” (Figure 9 and Figure 10 and Table 2).

## 4. Discussion

CMBs are recognized as important biomarkers for cerebrovascular diseases. The manual assessment is, however, time-consuming and error-prone. In the present cohort, a median of two CMBs per patient was observed, and counts of more than 10 CMBs are considered high [27]. From the example (Figure 1) given above, it is clear that the total number of CMBs can be considerably higher. This is why there is an ever-growing search for computer-aided detection systems that reliably detect CMBs with a low average count of false positives. Recent research has identified ways to obtain a high sensitivity with low false positive counts [13,21]. The problem with these approaches, however, is that they might not work as well across datasets with different MRI parameters, which is why we propose a method that can be trained on a small dataset with high sensitivity and a low false positive count. Using this two-stage approach with only 15 scans and 40 marked CMBs, we detect 95% of all CMBs while averaging 4.1 false positives per scan. When using a lower threshold, we reached a sensitivity of 97.5% with 14.5 false positives per scan on average. Our results show that it is possible to train neural networks on small datasets with high sensitivity and low false positive counts, which is advantageous in medical settings because radiological facilities can retrain the network using their own data and specifically adapt the network to their MRI sequences. A primary limitation of our current implementation is the increased computational processing time required to run the networks in parallel to generate the final results. With our hardware setup (see Section 2.3), the computation of the 3D U-Net prediction using the sliding window took approximately 20 min. Using both trained 3D U-Nets doubled that time. The processing time of the 3D CNN additionally took a couple of seconds. This processing time is much slower than the 64.3 s reported by Dou et al. [17] and the 20 s achieved by Chen et al. [28] with their workflow. However, this computational time could be improved by using two or more machines with GPU processing units, which would halve the time required for the sliding window 3D U-Net prediction. Further, increasing the 3D U-Net input and output volumes would also lead to faster processing times. Another shortcoming of our approach is the ability to detect CMBs in a cluster. Here, 3 of the 4 CMBs that were missed during validation had some low-intensity tissue near them, which led the 3D CNN to discriminate them. This behavior comes from training the 3D CNN with the U-Net mimics, which were often low-intensity vessels. Through these mimics, the 3D CNN learned to discriminate when points of low intensity were close by. The sensitivity could be improved by reducing the number of mimics during 3D CNN training, but this would also lead to an increase in false positives. It can be assumed that using this approach with a larger dataset would lead to a better detection of CMBs that occur in a cluster or have a low-intensity profile.

## 5. Conclusions and Future Work

We showed that it is possible to create a workflow with high sensitivity while using only a few training samples. Specified augmentation techniques (see Section 2.2.2) were used to increase the sample size. In order to counteract any problems that might arise as a result, such as the impossibility of a CMB occurring in this form of augmentation, the receptive fields (input layers) were enlarged to enrich the CMB excerpt with more contextual information. Furthermore, the results of the augmentations were constrained to the center of the receptive field, e.g., a shift of 1 to 2 voxels around the center. By ensuring that a CMB was centered, it became easier for the models to recognize a CMB. This approach allows smaller radiological facilities to train the networks using their own datasets, largely independent of their size. Even though the workflow performs well on a small dataset, it still requires further testing across other larger datasets to verify the performance when trained with different MR parameters. A larger dataset allows using only one 3D U-Net and only one 3D CNN rather than combining two different 3D U-Nets and CNNs. This would drastically speed up the process, making it more feasible for use in a clinical environment.

## Figures and Tables

**Table 1:** Comparison of various published studies. (FP avg: average number of false positives).

Year	FP Avg.	Sensitivity	Scans	Technical Approach	Paper
2011	107	81.70%	6	statistical thresholding algorithm and support vector machine (SVM) trained by supervised learning	Barnes et al. [14]
2012	17	71.20%	18	radial symmetry transform and manual discrimination	Kuijf et al. [15]
2012	4.1	91.00%	237	thresholding, ROI labelling and classification	Ghafaryasl et al. [16]
2012	2.74	93.16%	320	3D fully convolutional neural network for candidates and 3D convolutional neural network to discriminate between CMBs and mimics	Dou et al. [17]
2013	44.9	86.50%	15	2D fast radial symmetry transformation and 3D region growing on CMB candidates	Bian et al. [18]
2014	16.84	92.04%	41	cascade of random forest classifiers analyzing robust Radon-based features and manual discrimination	Fazlollahi et al. [19]
2016	13.9	90.80%	33	random forest classifier (based on SWI and T1), segmentation and object-based classifier	Van den Heuvel et al. [20]
2019	1.6	95.80%	195	candidate detection (3D fast radial symmetry transformation) and discrimination (deep residual neural network)	Liu et al. [21]
2020	1.42	93.60%	179	candidate detection (YOLO) and 3D-CNN	Al-Masni et al. [22]
2022	17.1	90.00%	45	classification Patch-CNN, Segmentation-CNN and U-Net	Koschmieder et al. [12]
2022	1.9	91.50%	265	segmentation (MiMSeg algorithm), filtration, filter extraction and CNN	Suwalska et al. [13]
2026	4.1	95.00%	15	advanced preprocessing, 3D U-Net first stage, 3D CNN discrimination stage	Our study (low FP)
2026	14.5	97.50%	15	advanced preprocessing, 3D U-Net first stage, 3D CNN discrimination stage	Our study (high sensitivity)

**Table 2:** 3D U-Net comparison. (FP avg: average number of false positives).

FP Avg.	Sensitivity	Batch Size
47.53	95.83%	20
38.73	96.67%	30
38.15	98.33%	combined

## Data Availability

Restrictions apply to the availability of these data. Data were obtained from [Public Repository, Department of Computer Science & Engineering, The Chinese University of Hong Kong] and are available [http://www.cse.cuhk.edu.hk/~qdou/cmb-3dcnn/cmb-3dcnn.html] (accessed 15 July 2022).

## Abbreviations

The following abbreviations are used in this manuscript:

CMB	Cerebral microbleeds
CNN	Convolutional neural network
ROI	Region of interest
SWI	Susceptibility-weighted imaging
YOLO	You Only Look Once
